# An Emerging Role of Micro- and Nanoplastics in Vascular Diseases

**DOI:** 10.3390/life14020255

**Published:** 2024-02-16

**Authors:** Seung Eun Lee, Hyun Kyung Yoon, Do Yun Kim, Taek Seung Jeong, Yong Seek Park

**Affiliations:** 1 Department of Microbiology, College of Medicine, Kyung Hee University, Seoul 02447, Republic of Korea; 2 Department of Biomedical Science, Graduate School, Kyung Hee University, Seoul 02447, Republic of Korea; yhkyhk2022@khu.ac.kr (H.K.Y.); 1018kdy@naver.com (D.Y.K.); jtaekseung@khu.ac.kr (T.S.J.)

**Keywords:** micro- and nanoplastics, environmental contaminants, toxicity, vascular diseases

## Abstract

Vascular diseases are the leading causes of death worldwide, and they are attributable to multiple pathologies, such as atherosclerosis, diabetes, and chronic obstructive pulmonary disease. Exposure to various environmental contaminants is associated with the development of various diseases, including vascular diseases. Among environmental contaminants, micro- and nanoplastics have gained attention as global environmental risk factors that threaten human health. Recently, extensive research has been conducted on the effects of micro- and nanoplastics on various human diseases, including vascular diseases. In this review, we highlight the effects of micro- and nanoplastics on vascular diseases.

## 1. Introduction

Vascular diseases are the leading causes of death worldwide. These include any condition that affects the circulatory system or blood vessels, ranging from diseases of the veins, lymph vessels, and arteries to blood disorders that affect circulation. Typically, vascular diseases begin with an injury to the endothelial wall, and adaptive and maladaptive remodeling of the vascular wall represent key disease processes [1,2]. In several vascular diseases, the central pathophysiological events include the stimulation of pro-inflammatory signaling pathways, expression of cytokines/chemokines, and promotion of oxidative stress [3,4,5].

The most important risk factors for vascular diseases include hypertension, diabetes, obesity, hyperlipidemia, and smoking. Vascular diseases have also been associated with environmental contaminants, one of the most important causes of mortality and disability worldwide [6]. Chowdhury et al. reported that exposure to environmentally toxic metals is associated with an increased risk of cardiovascular and coronary heart diseases [7].

Ambient air pollution is the most important global environmental risk factor contributing to human death [8,9]. A recent study evaluated the contribution of long-term exposure to air pollutants (PM2.5, CO, and NO_2_) to the pathogenesis of peripheral arterial occlusive disease [10]. Specifically, accumulating evidence has revealed that exposure to environmental contaminants, particularly fine particulate matter, is a major contributor to cardiovascular mortality and morbidity [11,12]. Therefore, in this narrative review, we focus on vascular diseases related to micro- and nanoplastic (MNP) exposure.

## 2. MNPs as Environmental Contaminants

Plastics have infiltrated several aspects of modern life, and they have been utilized in packaging, health care, construction, aviation, logistics, and clothing [13]. The growth in plastic usage can be attributed to its beneficial properties, such as low cost, strength, lightweight, durability, corrosion resistance, electrical insulation, and high thermal properties [14]. Global plastic production doubled from 2000 to 2019 to 460 million tons, and this trend is expected to continue [15]. Supply chain systems encompassing the production, utilization, management, and disposal of fossil-based plastics pose serious environmental challenges [16,17]. Among plastic wastes, biodegradable plastics have garnered public attention as an encouraging substitute for conventional non-degradable plastic polymers that generate serious plastic pollution, given that biodegradable plastics are susceptible to microbial biodegradation and are thus environmentally harmless [18,19,20,21,22,23].

### 2.1. MNPs

**Figure 1:**
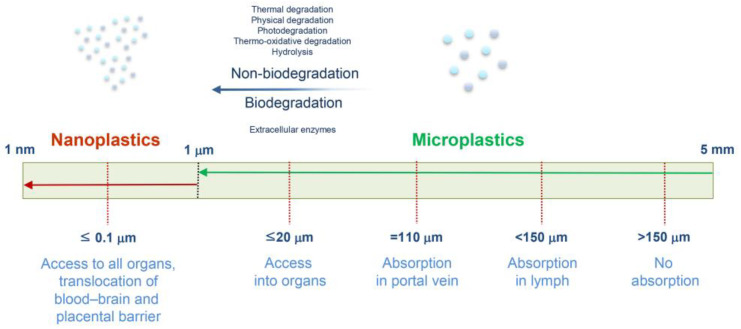
The fate of micro- and nanoplastics in mammalian bodies.

In 2004, the risks associated with MNPs were introduced, and their potential adverse effects on living organisms have recently attracted considerable attention [24,25,26]. After deposition in the environment, plastic waste is degraded into small fragments by exposure to ultraviolet (UV) radiation, oxidation, biodegradation, disintegration, and mechanical erosion, eventually resulting in MNPs [27,28]. Microplastics (MPs) are small artificial polymer particles (<5 mm) that are not the end products of plastic waste, given that they degrade into nanoplastics (NPs) (<1000 nm) [29,30] (Figure 1).

**Figure 2:**
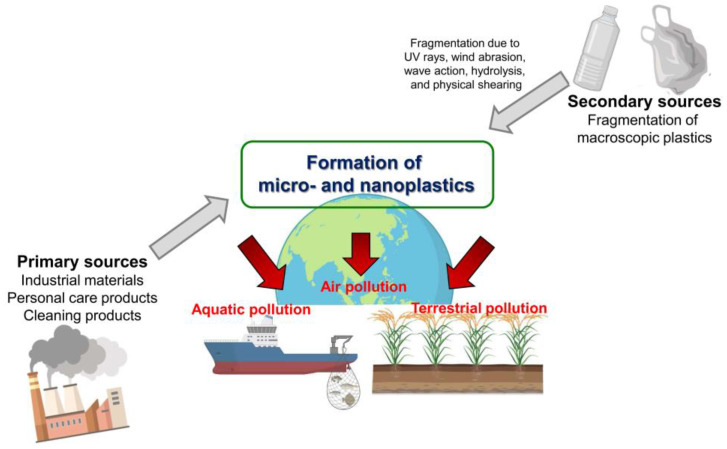
Sources of micro- and nanoplastics in the environment.

Currently, two types of MPs are recognized. Primary MPs contain plastic fragments or particles ≥ 5.0 mm in size before entering the environment. They originate from plastic pellets in the pre-production stage of the manufacturing industries, including plastic powder, plastic resin flakes, scrubbers, commercial cleaning abrasives, or tow used to generate plastic products [31]. Meanwhile, secondary MPs are generated by the degradation of larger plastic objects through natural weathering processes, such as atmospheric deposition, aquaculture, land-based sources, air transportation, and textile production, after entering the environment [32,33]. MPs are potential contaminants of emerging concern owing to their abundance in global environments, including terrestrial, atmospheric, and aquatic environments [34,35] (Figure 2).

### 2.2. Environmental Pollution with MNPs

Despite widespread awareness that the excessive usage and mismanagement of plastics are responsible for global environmental problems, global plastic waste production has more than doubled from 2000 to 2019 to 359 million tons. Furthermore, approximately 6.1 million tons of plastic waste is dumped in aquatic environments, with approximately 1.7 million tons presumed to have entered the oceans [36]. Under this ambitious scenario, Borrelle et al. have predicted that 20–53 million metric tons of plastic emissions/year will enter aquatic ecosystems by 2030 [37]. The presence of MPs in aquatic environments has been reported elsewhere as a global concern [38]. MPs have been found in abiotic and biotic areas of the ocean, including beaches [39,40], surface waters [41,42], sediment [43,44,45,46], fish [47,48,49], and shellfish [50,51].

Soil (such as home gardens, greenhouses, farmlands, and floodplain soils) is an enormous reservoir of accumulated MPs. Sewage sludge is among the several sources of MPs in the soil. Sewage-irrigated farmlands contain a significantly higher distribution of MPs compared with non-sewage-irrigated agricultural fields (5190 particles/kg vs. 2030 particles/kg) [52].

Furthermore, atmospheric pollution caused by airborne MPs is a growing concern worldwide. In recent years, airborne MPs have been discovered worldwide [53,54,55,56,57,58], with concentrations ranging from 0.01 MP m^−3^ (West Pacific Ocean) [59] to 5650 MP m^−3^ (Beijing, China) [60]. Airborne MPs primarily originate from textile fragmentation and decomposition processes, whereas nonfibrous particles predominantly arise from the degradation of packaging materials.

### 2.3. MNPs in the Food Chains and Food

The notable properties of plastics, such as strength, variability, flexibility, lightness, and persistence, make them suitable for packaging food and other goods [61]. As the use of plastics pervades various segments of the food chain, the instances of consumer exposure to MPs have increased considerably. MPs enter the human food chain mainly through contaminated foods and can affect human health [62]. Recent studies have reported an abundance of MPs in terrestrial food chains, including chicken stomachs (5.1 particles/g) and feces (105 particles/g) [26,63] and sheep feces (1000 particles/g) [64]. In addition, the presence of MPs has been documented in water, honey, beer, seafood, and sugar [65]. Overall, MNPs have the potential to be absorbed into the body through various stages of the food chain, circulate, and accumulate, and are thereby expected to have an adverse effect on the circulatory system. For instance, in vivo murine models exhibited that intestinal-delivered MPs reach the bloodstream within 15 min after ingestion, mainly accumulating in the liver [66,67].

## 3. MNP Toxicity in the Vascular System

### 3.1. Toxicities in Other Animal Cells

Florance et al. showed that anionic surfactant-stabilized NPs potently stimulate dysfunctional lipid homeostasis in brain- and tissue-specific macrophages in mice. The authors suggested that NPs were potent stimuli for foam cell formation and lipotoxicity, leading to the pathogenesis of atherosclerosis and posing a major risk to animal and human health [68].

A recent study examined the effects of MPs on endothelial and immune cells associated with vascular inflammation in mice. MP administration augmented the expression of adhesion molecules and cytokines in the aorta, indicating that MPs are a new environmental risk factor attributable to endothelial inflammation [69].

Furthermore, the in vivo effects of long-term MP exposure and potential exposure-induced kidney, liver, and cardiovascular toxicities have been investigated. Exposure to MPs aggravates vascular lesions and organ injuries, particularly in the heart, liver, and kidney. Based on RNA sequencing data, long-term exposure to MPs may be related to alterations in the biological features and global gene expression patterns in mice, providing novel insights into the biological toxicity of MPs [70]. N6-methyladenosine (m6A) modifications in ncRNAs have been identified as manipulating factors in cardiovascular diseases [71,72]. Zhang et al. demonstrated the N6-methyladenosine (m6A) modification profile of non-coding RNA (ncRNAs) in myocardial tissues exposed to MP using RNA sequencing (RNA-seq) and methylated RNA immunoprecipitation sequencing (MeRIP-seq). Differentially expressed ncRNAs were altered by MP exposure and were enriched in several cardiotoxicity-related signaling pathways. This study suggests that m6A modification of ncRNAs is involved in MP-induced myocardial damage [73].

Shiwakoti et al. have demonstrated the potential effects of NPs on the cardiovascular system at the cellular level. NP-exposed endothelial cells (ECs) could promote the acquisition of senescence markers; senescence-associated β-galactosidase activity; and p16, p21, and p53 protein expression, resulting in suppressed proliferation. Their findings support the effects and underlying mechanisms of NPs on the cardiovascular system and may provide pharmacological targets for the prevention of cardiovascular diseases [74]. Dhakal et al. demonstrated the role of sodium glucose co-transporter (SGLT2) in NP-induced endothelial senescence and dysfunction of porcine coronary arteries. NPs significantly upregulated SGLT2 and senescence-associated-β-galactosidase (SA-β-gal) activity, whereas suppression of SGLT2 significantly diminished SA-β-gal activity in porcine coronary artery endothelial cells (PCAEC). This study highlights the association of premature endothelial senescence by NPs with SGLT2 and provides a potential therapeutic target to prevent environmental pollutant-elicited cardiovascular diseases mediated by endothelial senescence and dysfunction [75]. Basini et al. revealed the potential effects of NPs on endothelial aortic cells (AOCs) by evaluating the ability of NPs to specifically interact with endothelial cells using fluorescence microscopy. NPs co-localize with AOC, increase AOC metabolic activity and vascular endothelial growth factor (VEGF) production, and disrupt the redox status [76].

Exposure to MPs can induce cerebral hemorrhage and promote the production of microthrombi and loss of Purkinje cells in chicken brain tissue. Moreover, a previous study elucidated the detailed mechanism underlying subsequent brain injury from the perspective of MP-induced physical injury (cerebral hemorrhage), thereby comprehensively clarifying MP exposure-induced neurotoxicity [77]. According to a study by Zhang et al., MPs can induce cardiotoxicity via ROS-driven NF-κB-NLRP3-GSDMD and the AMPK-PGC-1α axis in chickens. MP exposure triggered myocardial inflammation and pyroptosis through NF-κB-NLRP3-GSDMD axis- and AMPK-PGC-1α axis-mediated mitochondrial and energy metabolism dysfunction. The authors provided a better understanding of the potential role of MPs in MP exposure-induced cardiotoxicity [78].

Zhang et al. observed the effect of MPs on myocardial development in the primary cardiomyocytes of chicken embryos. Histopathological observations showed that MPs had a loose and irregular myocardial arrangement, large cell gaps, and broken myocardial fiber bundles. These data suggest that MP-induced myocardial dysplasia in birds is principally attributed to the ER stress-mediated autophagic pathway [79].

Moreover, the effects of MPs on cardiac tissues and the mechanisms of pyroptosis and oxidative stress during cardiomyocyte injury have been reported in Wistar rats. Administration of MPs can lead to apoptosis and pyroptosis, as evidenced by the stimulation of interleukin (IL)-1β and IL-18 expression. These findings illustrate that pyroptosis plays a crucial role in MP-induced cardiotoxicity, which may help clarify the mechanism underlying MP exposure-induced cardiac dysfunction [80]. Roshanzadeh et al. showed that MPs can reduce the contractility of cardiomyocytes by influencing intracellular Ca^2+^ level, electrophysiological activity, mitochondrial membrane potential, and cell metabolism of neonatal rat ventricular cardiomyocytes (NRVMs) [81].

Zhao et al. revealed that consumption of MPs promotes adiposity, hyperglycemia, and changes in the gut microbiome in mice. As the major finding, the authors noted that MP ingestion could promote increased obesity in mice in a dose- and size-dependent manner. These results suggest that the ingestion of MPs stimulates a cardiometabolic disease phenotype and thus may be considered a new risk factor for cardiovascular disease [82]. Wang et al. demonstrated that NPs in the blood and aorta of ApoE^−/−^ mice aggravate the artery stiffness and enhance atherosclerotic plaque formation. NP exposure stimulates phagocytosis by M1 macrophages, interrupts lipid metabolism, and increases long-chain acyl carnitines (LCACs). This study provides new insights into the mechanisms underlying MNP-induced cardiovascular toxicity [83]. In vivo studies by Wang et al. demonstrated that NPs can induce vascular injury, structural damage to vascular endothelial cells, and inflammatory responses. Structural damage and dysfunction of vascular endothelial cells upon exposure to NPs occur through the activation of the Janus kinase 1/signal transducer and activator of the transcription-3/tissue factor (JAK1/STAT3/TF) signaling pathway [84]. Zhang et al. evaluated the cardiotoxicity induced by respiratory exposure to NPs, including the accumulation of NPs, histological observation, assessment of cardiac function, detection of biomarkers, and transcriptomic studies, and showed that NP exposure induced acute cardiotoxicity in a dose- and time-dependent manner [85]. Shan et al. found that NPs could accumulate in the brains of mice, promptly activate microglia, and cause damage to neurons. Moreover, NPs could accumulate in human cerebral microvascular endothelial cell (hCMEC/D3) cells and stimulate nuclear factor kappa-B (NF-κB) activation, ROS generation, necroptosis, and inhibition of transendothelial electrical resistance (TEER) and occludin expression. These findings suggest that NPs stimulate the inflammatory response, oxidative stress, and necroptosis in hCMEC/D3 cells, which may also contribute to the impairment of tight junctions (TJs) in the blood–brain barrier (BBB) [86].

A recent study has provided insights into the role of MPs as potential mediators of unbridled oxidative imbalance and dyslipidemia in mammals. Oral administration of MPs lowered high-density lipoprotein cholesterol and elevated low-density lipoprotein cholesterol levels. In addition, the authors observed that MP-exposed rats exhibited sex-based differences in atherogenic indices. These data indicate that dietary MPs may lead to dyslipidemia, and male rats have a high cardiovascular risk [87].

The effects of inhaled MNPs on the cardiovascular system have been previously reported in rats. Cardiovascular function was assessed by measuring mean arterial pressure (MAP), wire myography of the aorta and uterine artery, and pressure myography of the radial artery. MNP inhalation induces various adverse cardiovascular dysfunctions such as increased blood pressure and diminished uterine vascular dilation. These data suggest that the inhalation of MNP aerosols leads to alteration of inflammatory, cardiovascular, and endocrine activities [88].

Li et al. revealed that MPs could induce cardiomyocyte apoptosis and activate Wnt/β-catenin signaling via oxidative stress induction, leading to heart fibrosis and cardiac dysfunction in rats. These findings provide novel evidence for a potential mechanism of MP-induced cardiovascular toxicity [89]. Yan et al. demonstrated that MP exposure elicited mild vascular calcification (VC) in normal murine and aggravated VC in vitamin D3 plus nicotine (VDN)-treated murine. Their work revealed that the mechanism of vascular injury by MPs may involve the induction of intestinal mucosal barrier dysfunction and an inflammatory response in the body. These observations present a theoretical basis for the risk assessment of MP-induced cardiovascular diseases [90].

The potential toxic effects of MNP exposure were estimated in zebrafish embryos at various developmental stages. Exposure to MNPs impairs intact development of the caudal vein plexus, leading to prematurity via pathological angiogenesis. These findings indicate that exposure to MNPs can lead to developmental disorders with substantial growth impairment and peripheral microcirculation dysfunction, and thus provide new insights into the use of plastics [91]. Sun et al. found that NPs could induce pericardial edema, restrain sub-intestinal angiogenesis, lead to endothelial cell dysfunction, and simultaneously obstruct CO and blood flow velocity in zebrafish embryos. These findings suggest that NPs can elicit cardiovascular toxicity in zebrafish embryos, thereby providing essential information for the safety assessment of NPs [92].

Pitt et al. investigated the potential toxicity of NPs during the development of zebrafish (*Danio rerio*). The authors observed that NPs accumulated in the yolk sac as early as 24 h post-fertilization (hpf), migrated to the gallbladder, gastrointestinal tract, pancreas, liver, heart, and brain throughout development (48–120 hpf), and diminished during the depuration phase (120–168 hpf) in all organs. Notably, NPs were localized in the pericardium only at 1 and 10 ppm; however, a significant decrease in heart rate was observed even when larvae were exposed to 0.1 ppm NP, indicating that NPs can induce cardiotoxicity even at a low concentration. Collectively, these data demonstrated that NPs may produce organ toxicity specific to their developmental distribution patterns [93]. A recent study demonstrated that exposure to NPs can prompt size- and dose-dependent effects on neurobehavior and potential hepatic, cardiovascular, gastrointestinal, epigenetic, and metabolic dysfunction, as determined by transcriptomic analysis of NP-exposed zebrafish [94]. Dai et al. evaluated the significant developmental toxicity of NPs in zebrafish embryos. Developmental impairments were observed in zebrafish embryos following exposure to various NP concentrations. Additionally, NP exposure led to vascular malformation by modulating VEGFA/VEGFR pathway-correlated gene expression during the early developmental stages of zebrafish. Thus, their findings demonstrated the toxicological effects of NPs on the development of the cardiovascular system [95]. Santos et al. investigated vasotoxicity, developmental toxicity, cytotoxicity, and behavioral impairments in zebrafish induced by NPs exposure. The authors assessed the cardiotoxicity, neurotoxicity, teratogenic effects, and larval morphological changes caused by NPs using the zebrafish embryo–larval toxicity test (ZELT). This study showed the harmful effects of NPs on the early developmental stages of fish, highlighting their environmental risk [96].

Wu et al. demonstrated the adverse effects of NPs on cardiac apoptosis and inflammation in carp, mediated by an oxidative stress-induced Th1/Th2 imbalance and activation of the IGFBP3/p53/ACHE signaling pathway. The authors provided a theoretical basis for toxicological research on MNPs in aquatic animal hearts [97].

### 3.2. Toxicities in Human Cells

In humans, the routes of MNP exposure include ingestion of plastic-contaminated food and water or inhalation of plastic-contaminated air [54,55]. Consequently, the digestive and respiratory systems are the primary sites of contact for MNPs. MNPs produce systemic toxicity by penetrating the cell membranes and causing cellular internalization.

Rotchell et al. examined digested human saphenous vein tissue samples using μFTIR spectroscopy and reported the first evidence of MP contamination in human vascular tissues [98]. Sun et al. qualitatively and quantitatively identified the presence of various types of MPs in the human endometrium using laser direct infrared (LDIR) spectroscopy [99]. These studies provide a prospective direction for future biological and environmental studies.

Lee et al. reported that MP exposure is a noteworthy risk factor for endothelial dysfunction and vascular malformations. Prolonged MP exposure can lead to reduced cell viability associated with the stimulation of autophagy/necrosis. Thus, the authors provided considerable evidence regarding the possible threat posed by MPs to human vascular system homeostasis [100].

A recent study has explored the interaction between low levels of MNPs and human umbilical vein endothelial cells (HUVECs) as a model of vascular endothelial cells. In this study, no remarkable changes in inflammation, autophagy, reactive oxygen species (ROS) levels, lactate dehydrogenase release, or adhesion molecule expression at exposure concentrations ranging from 5 to 25 μg/mL were detected. However, cell viability declined at extremely high exposure (100 μg/mL). These findings contribute to further risk assessments of MNPs in human health, including the vascular endothelium [101]. Moreover, NP-stimulated vasculature permeability is primarily biophysical/biochemical in nature and is not correlated with cytotoxic events, including ROS production, apoptosis, and autophagy [102]. Lu et al. demonstrated that smaller NPs (100 nm) result in cell membrane damage, autophagosome formation, and autophagic flux blockage in HUVECs. This study provided new insights into the potential effects of NPs on human vascular endothelial cells, which is a crucial contribution to the health risk assessment of NPs in the vascular system [103].

MPs can induce endothelial cell (EC) dysfunction by impairing mitochondrial function and upregulating pro-inflammatory cytokines, thereby triggering apoptosis. In addition, MPs induce atherosclerotic plaque formation and vascular inflammatory responses in vivo. Moreover, correlation analysis between transcriptomics/metabolites and cell injury has been reported. These findings indicate that MPs prompt abnormal transcriptomic and metabolomic responses in ECs both in vitro and in vivo, and these results may be utilized in the development of effective preventive strategies against MP damage [104]. Additionally, Fu et al. revealed that NPs induce oxidative stress and damage the mitochondria membrane potential in HUVECs. NPs dysregulate mitochondrial dynamics, function-related gene expression, and replication. Results showed that NPs were cytotoxic in HUVECs, as evidenced by reduced cell viability, diminished mitochondrial membrane potential, and augmented ROS generation, which provides direct evidence of the potential risks of NPs [105].

Chen et al. introduced an optically assisted thrombus platform to reveal interactions between MPs and the vascular system. According to the on-chip results, MP invasion decreased fibrin binding to platelets (*p*-value < 0.0001) and showed a high risk of thrombus shedding into the circulation compared to that in normal thrombus. Accordingly, they demonstrated the mechanism of thrombus invasion mediated by MPs using a microfluidic platform, suggesting a potential method for further exploration of MP-related health concerns [106].

Zhou et al. utilized a human pluripotent stem cell-derived three-dimensional cardiac organoid (CO) model to explore the adverse effects of MP exposure on the human heart in vitro. MPs augmented the inflammatory response, oxidative stress, collagen accumulation, and apoptosis, which are associated with increased expression of the cardiac-specific markers MYL2, MYL4, and CX43 in COs [107].

MPs have been shown to induce oxidative stress by reducing antioxidant expression in human EA.hy926 vascular endothelial cells. Exposure to MPs resulted in vascular barrier dysfunction and altered apoptotic cytotoxicity. These data provide insights into the potential toxicity risks of MPs in the human circulatory system [108].

Florance et al. reported that NPs induce lipid droplet accumulation and acute mitochondrial oxidative stress in humans. Accumulated lipid droplets are further delivered and accumulate in lysosomes, leading to impaired lysosomal clearance. The authors suggested that exposure to NPs is a potent stimulus for dysregulated macrophage foam cell formation and lipid metabolism, a characteristic feature observed during atherosclerosis, thereby posing a severe risk to human health [109].

Plastic particles such as MNPs enter the body through multiple pathways involving dermal areas, inhalation, and ingestion, and are transported via the bloodstream. Therefore, understanding the correlation between pollutants such as MNPs and blood cells might have significant implications for the pathophysiology of various human diseases. Recent studies revealed that aggregation and distribution of MNPs in blood are associated with the bioavailability, inflammatory, and immune responses of multiple cells such as monocytes, leukocytes, erythrocytes, and endothelial cells in the human body [110,111,112,113,114].

**Figure 3:**
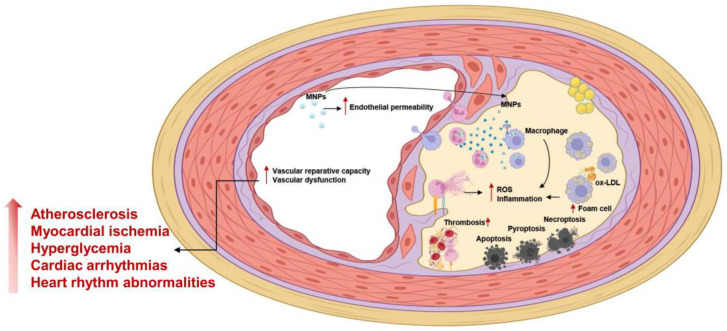
The possible influence of micro- and nanoplastics on the vascular system.

Recently, MNPs have emerged as a serious hazard in the environmental and food safety fields, causing cellular damage and affecting cell survival, death, apoptosis, inflammation, and various immune responses. Overall, this review focused on discussing the association between MNP exposure and vascular diseases (summarized in Table 1 and Table 2 and Figure 3).

## 4. Conclusions

Plastic production worldwide has increased 20-fold compared to its initial value, owing to its ease of use, cost-effectiveness, and durability. The most commonly distributed plastics in the environment are polyvinyl chloride, polyethylene, polypropylene, polyethylene terephthalate, and polystyrene [115]. Global concerns about plastic-induced environmental pollution have increased substantially. Plastic waste is exposed to the environment and forms MPs through mechanical abrasion, ultraviolet (UV) irradiation, and biological weathering. MPs have been found in every component of nature, including rivers, lakes, oceans, soil, atmosphere, and the Antarctic Ocean [116]. MPs can be attributed to multiple sources, including primary (synthetic materials) and secondary sources (derived from the breakdown of larger plastic particles). MNPs have been shown to negatively affect animal growth, reproduction, and metabolism through several mechanisms, including ingestion and bioaccumulation. Furthermore, MPs can induce oxidative stress, DNA damage, histopathological damage, intestinal microflora dysbiosis, neurotoxicity, genotoxicity, reproductive toxicity, and metabolic disorders, which are primarily governed by the MP type, size, shape, and dosage.

Overall, this review provides a comprehensive overview of the current knowledge regarding the effects of MNPs on the structure and function of the vascular system and their impact on the pathophysiology of vascular disorders. A better understanding of the roles of MNPs will broaden our understanding of cellular systems.

## Figures and Tables

**Table 1:** Summary of micro- and nanoplastic toxicity on the vascular system.

Models	Type and Size of Particles	Conc.	Modes of Action	References
RAW 264.7 and BV2 microglial cells	Polystyrene (PS) NPs 0.20 μm	50, 100, and 200 μg/mL	Disruption of lipid metabolism	Florance et al., 2021 [68]
Murine myocardial endothelial cells	PS MPs 1 μm	0.54 ng/mL, 54 ng/mL, and 5.4 μg/mL	Induction of endothelial activation and monocyte adhesion	Vlacil et al., 2021 [69]
Porcine coronary artery endothelial cells (PCAECs)	PS NPs 25 nm	0.1, 1, and 10 μg/mL	Induction of premature EC senescence	Shiwakoti et al., 2022 [74]
	PS NPs	5 and 10 μg/mL	Induction of premature EC senescence and dysfunction	Dhakal et al., 2023 [75]
Porcine aortic endothelial cell line (AOC)	PS NPs 100 nm	5, 25, and 75 μg/mL	Alteration of AOC metabolic activity, redox status, and VEGF production	Basini et al., 2023 [76]
Chicken	PS MPs 5 μm	1, 10, and 100 mg/L in drinking water	Alteration of intracerebral hemorrhage and mitochondrial dysfunction	Yin et al., 2022 [77]
	PS MPs 5 μm	1, 10, and 100 mg/L in drinking water	Induction of myocardial inflammation and pyroptosis	Zhang et al., 2022 [78]
Bird	PS MPs 5 μm	1, 10, and 100 mg/L in drinking water	Induction of endoplasmic reticulum (ER) stress in the myocardium	Zhang et al., 2022 [79]
Mouse	MPs 5 μm	1000 μg/L in drinking water	Alterations in biological features and global gene expression patterns	Shi et al., 2022 [70]
	PS MPs 10 μm	1000 μg/L in drinking water	Induction of m6A modifications of ncRNAs	Zhang et al., 2023 [73]
	PS NPs 0.5 and 5 μm	0.1 and 1.0 μg/mL in drinking water	Induction of adiposity and hyperglycemia	Zhao et al., 2022 [82]
	PS NPs 50 nm	2.5, 25, and 250 mg/kg	Induction of MARCO upregulation, macrophage activation, and lipid metabolism disruption	Wang et al., 2023 [83]
	PS, PS-NH_2_, and PS-COOH NPs 50 nm	0.05, 0.5, 5, 10, and 20 mg/kg	Induction of injury and dysfunction through the activation of the JAK1/STAT3/TF pathway	Wang et al., 2023 [84]
	PS NPs 40 nm	7.9 × 10^12^, 1.85 × 10^13^, and 4.6 × 10^13^ items/m^3^	Induction of acute cardiotoxicity	Zhang et al., 2023 [85]
	PS NPs 42 nm	0.5, 2.5, 10, and 50 mg/kg	Induction of microglia activation and neuron damage	Shan et al., 2022 [86]
Rat	PS MPs 0.5 μm	0.5, 5, and 50 mg/L in drinking water	Induction of pyroptosis of cardiomyocytes and oxidative stress/inflammation	Wei et al., 2021 [80]
	Amine-modified PS NPs 50 nm	25 μg/mL	Reduction of collective contractility	Roshanzadeh et al., 2021 [81]
	PS MPs 5 mm	Feed containing 1, 5, and 10%	Alteration of dyslipidemia and oxidative imbalance	Nnoruka et al., 2022 [87]
	Polyamide MNPs with a median diameter of 2.81 μm	Exposure concentration of near 10 mg/m^3^	Alteration of inflammatory, cardiovascular, and endocrine activity	Cary et al., 2023 [88]
	PS MPs 0.5 μm	0.5, 5, and 50 mg/L	Alteration of cardiac fibrosis and dysfunction	Li et al., 2020 [89]
	PS MPs 5 μm	0.5 mg/L	Induction of mild vascular calcification (VC)	Yan et al., 2023 [90]
Zebrafish embryos	PS NPs, PS MPs 0.4 and 1 μm	0.00075–0.006%	Induction of developmental toxicity and microcirculation dysfunction	Park et al., 2022 [91]
	Polyethylene NPs 191.10 ± 3.13 nm	25, 50, 100, 200, 400, 600, 800, and 1000 μg/mL	Alteration of endothelial damage and hemodynamics changes	Sun et al., 2021 [92]
	PS NPs 20 nm	2, 5, and 8 mg/L	Induction of various vascular malformations	Dai et al., 2023 [95]
	PS NPs 23.03 ± 0.266 nm	0.04 and 34 ng/L, 34 μg/L	Induction of neurotoxicity and cardiotoxicity	Santos et al., 2024 [96]
Zebrafish (Danio rerio)	PS NPs 51 nm	0, 0.1, 1, and 10 ppm	Induction of cardiotoxicity	Pitt et al., 2018 [93]
	PS NPs 50 and 200 nm	0, 10, 100, 1000, and 10,000 ppb	Alteration of behavioral response (swimming hyperactivity)	Pedersen et al., 2020 [94]
Carp	PS NPs 50, 100, and 400 nm	1000 μg/L	Induction of cardiomyocyte apoptosis and myocardial inflammation	Wu et al., 2022 [97]
Human umbilical vein endothelial cells (HUVECs)	PS MPs 0.5, 1, and 5 μm	0, 20, 40, 60, 80, and 100 μg/mL	Reduction of viability and stimulation of autophagy/necrosis	Lee et al., 2021 [100]
	PS MNPs 1 μm	0, 5, 10, and 25 μg/mL	Reduction of cell viability	Lu et al., 2023 [101]
	PS, NH2-PS, PMMA NPs 30 and 50 nm	0.05 and 0.5 mg/mL	Induction of endothelial leakiness	Wei et al., 2022 [102]
	PS NPs 100 and 500 nm	0, 5, 10, 25, 50, and 100 μg/mL	Induction of autophagosome formation and autophagic flux blockage	Lu et al., 2022 [103]
	PS MPs 20, 50, 100, and 500 nm, 5 and 10 μm	1000 μg/mL	Alteration of endothelial dysfunction	Zhang et al., 2022 [104]
	PS NPs and NH_2_-PS NPs 50 nm	5, 10, 15, 20, and 25 μg/mL	Induction of oxidative stress	Fu et al., 2022 [105]
HUVECs and blood (human/mouse)	PS MPs 1 μm	0.1 and 1 μg/mL	Increased risk of thrombosis	Chen et al., 2022 [106]
Human embryonic stem cell line H1 (H1 ES)	PS MPs 1 μm	0, 0.025, 0.25, and 2.5 μg/mL	Increased the expression levels of cardiac-specific markers MYL2, MYL4, and CX43	Zhou et al., 2023 [107]
Human vascular endothelial EA.hy926 cells	PS MPs 2.2–6.5 μm	4 × 10^−6^–40 μg/mL	Induction of oxidative vascular cytotoxicity	Chen et al., 2023 [108]
THP-1 (human monocytes) and RAW 264.7 (murine macrophages)	PS NPs 0.2 μm	100 µg/mL	Dysregulation of lipid metabolism homeostasis	Florance et al., 2022 [109]

**Table 2:** Summary of micro- and nanoplastic effects on human vascular system.

Specimen	Type and Size of Particles	Effects on Vascular System	Reference
Human serum	PS NPs 80–170 nm	Internalization into granulocytes, monocytes, and myeloid dendritic cells	Mohr et al., 2014 [110]
Human whole blood	PS NPs 0.05–0.1 μm	Internalization into monocytes and white cells, genotoxic effects on monocytes and polymorphonuclear leukocytes	Ballesteros et al., 2020 [111]
Human peripheral blood mononuclear cells	PS NPs, PS MPs 20, 100, 200, 500, and 1000 nm	Internalization into phagocytes and macrophages, IL-6 secretion	Prietl et al., 2014 [112]
Human plasma	PS NPs 100 nm	Erythrocytes and lymphocytes inducing cytotoxicity, hemolysis, and genotoxicity	Gopinath et al., 2019 [113]
Human red blood cells	PS NPs 49.9 ± 6.3, 107.9 ± 1.4, and 243 ± 3.0 nm	Adhesion and aggregation to endothelial cells	Barshtein et al., 2016 [114]

## Data Availability

Not applicable.
